# Atorvastatin Exerts Context-Dependent Metabolic and Beneficial Effects on Behavior and Apoptotic Signaling in a Cafeteria Diet–Induced Metabolic Dysfunction Model

**DOI:** 10.1007/s11064-026-04874-9

**Published:** 2026-08-29

**Authors:** Ítalo Leonardo Diogo, Grazielle Aparecida Silva Maia, Israel José Pereira Garcia, Herica de Lima Santos, Vanessa Faria Cortes, Leandro Augusto Barbosa, Valéria Ernestânia Chaves, Luciana Estefani Drumond Carvalho

**Affiliations:** 1 Laboratório de Bioquímica Celular, Universidade Federal de São João Del Rei, Divinópolis, Minas Gerais 35501-296 Brazil; 2 Laboratório de Fisiologia, Universidade Federal de São João Del Rei, Divinópolis, Minas Gerais 35501-296 Brazil

**Keywords:** Obesity, Cafeteria diet, Atorvastatin, Memory, Apoptosis and neuroprotection

## Abstract

The growing prevalence of obesity has been associated with metabolic and neurobiological alterations that compromise brain integrity and behavior. Diet-induced obesity, often accompanied by hypercholesterolemia, activates apoptotic signaling pathways in the central nervous system, contributing to cognitive and behavioral impairments. This study evaluated the effects of atorvastatin on behavioral performance and apoptotic signaling pathways in the hippocampus and cerebral cortex of rats subjected to a hypercaloric cafeteria diet. Male Wistar rats were assigned to four groups: control diet (CTR), control diet plus atorvastatin (ATOR), cafeteria diet (CAF), and cafeteria diet plus atorvastatin (CAF+ATOR), and exposed to the diets for 24 days. Body weight, adiposity, and circulating cholesterol levels were measured to validate the metabolic model. Behavioral assessments included locomotor activity, immobility time, and recognition memory. The expression of pro-caspase−8, pro-caspase−9, and apoptosis-inducing factor (AIF) was quantified in frontal cortex and hippocampus. The cafeteria diet increased adiposity and circulating cholesterol levels, confirming metabolic dysfunction, whereas atorvastatin reduced cholesterol levels and prevented retroperitoneal fat accumulation. In addition, the cafeteria diet altered the expression of apoptosis-related proteins in the hippocampus and cortex, whereas atorvastatin attenuated these changes. Cafeteria-fed rats showed reduced exploratory activity and increased immobility. Spatial memory was impaired by both atorvastatin and the cafeteria diet but preserved in the CAF+ATOR group. Novel object recognition was impaired only in the ATOR group. These findings indicate that atorvastatin exerts context-dependent effects under conditions of diet-induced metabolic dysfunction.

**Graphical Abstract:**

**Figure:**
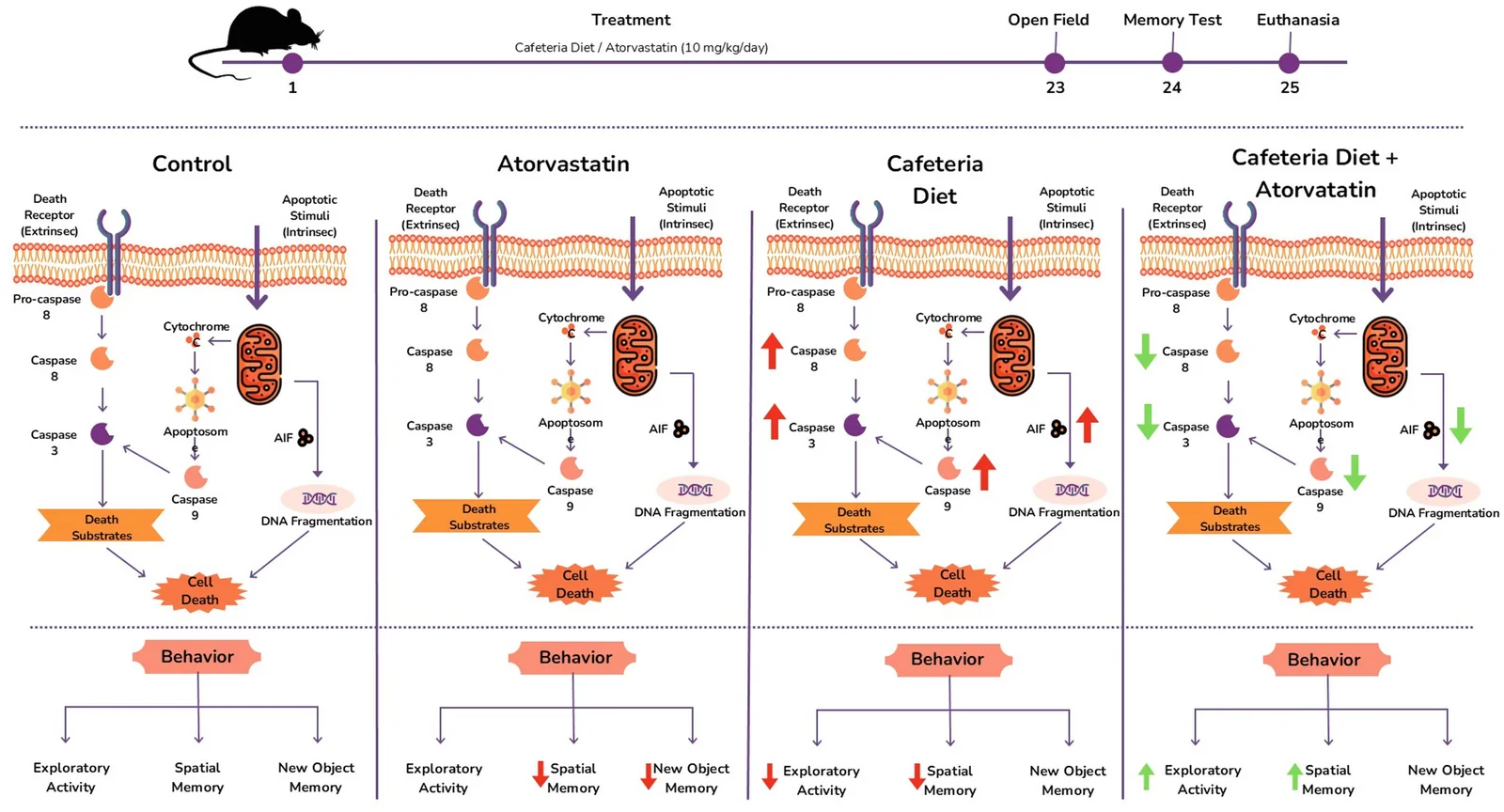
Experimental timeline and proposed mechanisms underlying the effects of atorvastatin treatment in cafeteria diet-induced obesity. Cafeteria diet-induced obesity promoted hypercholesterolemia, increased adiposity, behavioral impairments, and alterations of intrinsic and extrinsic apoptotic pathways in the hippocampus and cerebral cortex of rats. Atorvastatin treatment attenuated apoptotic signaling, reduced metabolic dysfunction, and improved exploratory and memory-related behavioral alterations, indicating neuroprotective effects under diet-induced metabolic stress. The figure was created using Canva (Canva Pty Ltd., Sydney, Australia).

**Supplementary Information:**

The online version contains supplementary material available at https://doi.org/10.1007/s11064-026-04874-9.

## Introduction

Obesity is a global public health problem, characterized by the accumulation of fat in the body [1]. According to the definition given by the World Health Organization (WHO), obesity refers to the excess or abnormal accumulation of fat in the body, which endangers health. This condition has been observed since ancient times, with Hippocrates emphasizing that obesity is not only a disease, but also a factor that predisposes to other health problems [2].

Furthermore, obesity interferes with the function of the nervous system. Obese children and young people have more behavioral problems and are more likely to develop psychopathologies, higher levels of anxiety, depression and stress [3]. Obesity considerably affects the exploratory capacity and cognitive functions of rats, mainly due to the loss of synapses, dysfunction of glial cells and increased brain inflammation [4]. Hippocampal formation is one of the brain regions most vulnerable to nutritional imbalances that occur in cases of obesity, and consequently causes deterioration in spatial learning, memory and changes in anxiety-related behavior in both the short and long term [5].

Studies with rats fed diets high in sugar, high in fat [6] or the cafeteria diet, which is high in calories and high in fat [7] have been used as experimental models for obesity studies. The cafeteria diet accurately reflects the high palatability and high energy density factors associated with the current obesity pandemic and prevalent in Western society [8]. In our model, rats fed the cafeteria diet have an increase of approximately 40% in energy intake and lipids contribute 20 ± 1%, carbohydrate 65 ± 1% and protein 15 ± 1% of the energy intake, compared with 12, 63, and 25%, respectively, in rats fed the control diet [9]. Cafeteria does not change the body weight, but increase the adiposity, evaluated by weight of retroperitoneal, inguinal, mesenteric and epididymal adipose tissue and lipid content of carcass and liver [10–12]. Previous study shows that young adult rats fed the cafeteria diet increase the brown adipose tissue thermogenesis and do not have changes in body weight [13].

The molecular changes caused by an excessive supply of nutrients are linked to increased production of reactive oxygen species (ROS) in cells, which can cause mitochondrial dysfunction and further raise ROS levels. The resulting accumulation of ROS in the respiratory chain triggers oxidative stress, which in turn is capable of causing apoptosis in various tissues, especially in the central nervous system [14].

Apoptosis, or programmed cell death, follows a well-organized sequence of events initiated by two primary pathways: an extrinsic pathway mediated by receptors, and an intrinsic pathway mediated by the mitochondria. Both pathways involve proteolytic and nucleolytic activity [15, 16].

Apoptosis is essential for maintaining adipose tissue homeostasis. Disruption of apoptotic processes in this tissue can lead to various disorders, including cancer, neurodegenerative diseases and diabetes [17, 18]. Adipokines such as leptin, resistin, adiponectin and TNFα can cross the blood–brain barrier or influence its function, modulating neuroinflammation and oxidative stress [19, 20].

Obesity is associated with hippocampal neurodegeneration and cognitive deficits, often mediated by chronic inflammation in adipose tissue, which triggers neuroinflammatory responses in the hippocampus [20, 21]. High levels of visceral adipose tissue are negatively associated with hippocampal volume and cognitive performance [22]. Alterations related to excessive adipose tissue accumulation can disrupt the blood–brain barrier, impair synaptic plasticity and hinder neurogenesis in the hippocampus. In addition, they can contribute to a reduction in the volume of grey matter in the hippocampus, impairing the areas responsible for learning, memory and cognition [19].

In an effort to control the deleterious effects of obesity, which leads to an increase in circulating cholesterol levels, the use of drugs has become increasingly common, including statins [23]. Atorvastatin is indicated for the treatment of hypercholesterolemia alone or in combination with hypertriglyceridemia and/or reduced blood levels of HDL; including those transmitted genetically/familiarly, when the response to diet and other non-pharmacological measures is inadequate [24].

Statins are known to have effects on the nervous system, improving memory impairment and neurotoxicity in diabetic mice [25], decreasing neuroinflammation in an epilepsy model [26] and inhibiting apoptosis in the hippocampus in an experimental Alzheimer’s model [27].Treatment with simvastatin in adolescent rats induced by a high-fat diet (HFD) was able to attenuate depressive behavior and reduce neuroinflammatory factors in the hippocampus [28]. In in vivo experiments, atorvastatin reduced the size of cerebral infarcts, improved memory and learning ability, reduced the amount of TUNEL-labelled neurons, suppressed the expression of cleaved caspase−3 and Bax/Bcl2, as well as activating the cAMP/PKA/p-CREB/BDNF pathway in the cerebral cortex [29]. Atorvastatin is described as a drug that effectively improves behavioral disorders [26, 30].

Therefore, this study was designed to evaluate the effects of atorvastatin in behavioral and biochemical parameters in the hippocampus and cortex of cafeteria-diet fed rats.

## Materials and Methods

### Animals

Male Wistar rats, approximately 28 days old and weighing 45–60 g at the beginning of the experimental protocol, were supplied by the Central Animal Facility of the Federal University of São João Del Rei-UFSJ. The animals were received after weaning (21 days of age) and acclimatized for 7 days in the sectoral animal facility (CT-INFRA II) before the experiments began. In the vivarium, the animals were kept in an environment with a 12-h light–dark cycle and controlled temperature (22 ± 2ºC). Efforts were made to avoid any unnecessary distress to the animals, and the lowest possible number of animals was used. A total of 40 rats were used in this study. The sample size for each experiment is indicated in the corresponding figure legends. All procedures were approved by the Ethical Committee for Animal Research of Federal University of São João Del Rei-UFSJ (Protocol n. 028/2018).

### Cafeteria Diet and Atorvastatin Treatment

Regarding cafeteria diet, over the course of 24 days, the rats were offered a cafeteria-type hypercaloric diet, obtained by supplementing a commercial balanced diet with various palatable ingredients, all of which had a higher calorie density than the commercial diet [31, 32]. The cafeteria diet consisted of a standard balanced diet [Nuvilab CR1, Nuvital, Brazil (22% protein, 55% carbohydrate, and 4.5% lipid)] supplemented each day with 4 different lipid-rich palatable items selected from a list of 12 (bacon, caramel candy, cashew nut, cookies, cornstarch biscuit, cheese biscuit, chocolate roll, chocolate wafers, nougat, peanut candy, potato chips, and toast). The details are available in Supplementary Data N°1. In addition, the water offered to these rats contained 20% sucrose. Control rats were fed the commercial diet only and consumed water ad libitum.

The treatment lasted 24 days, the rats received saline 0.9% or atorvastatin 10 mg/Kg/day, by gavage, according to the sample group. Throughout the treatment period, animals were monitored daily for signs of distress, weight loss, dysphagia, respiratory abnormalities, oral bleeding, or other clinical signs suggestive of gavage-related injury. To minimize procedural stress, all animals, including controls, underwent the same handling procedure. The animals were divided into the following experimental groups: I. CTR: fed a balanced diet and treated with saline; II. ATOR: fed a balanced diet and treated with Atorvastatin; III. CAF: fed a cafeteria diet and treated with saline and IV. CAF+ATOR: fed a cafeteria diet and treated with Atorvastatin.

After daily weighing and behavioral tests, the rats were euthanized by guillotine decapitation. The brain was then rapidly removed, and the hippocampi and cerebral cortex were carefully dissected, immediately frozen, and stored at − 80 °C until biochemical analyses. In addition,epididymal, retroperitoneal fat and blood was collected. The timeline of the experimental protocol can be seen in the figure abstract.

### Body Weight and Fat

All the animals were weighed daily to determine body weight variation. A weight variation curve over time was established and the final weight of each animal was subtracted from the initial weight, to determine the final weight gain. Immediately after euthanasia, the animal’s body was dissected and the epididymal and retroperitoneal fat were collected and weighed, according to Chaves’s method [9]. Data were expressed in grams.

### Total Cholesterol Quantification

On the day of euthanasia, the animals’ blood was collected in a buffered tube and carefully homogenized by inversion, about 5 times. The samples were stored on ice and ~30 min after collection, they were centrifuged in a refrigerated centrifuge (2 to 8 °C) at 2000 g for 10 min. After separation, the plasma was collected and stored in microtubes and the pellet discarded. The concentration of cholesterol was determined by an enzymatic colorimetric method, using the Cholesterol Monoreagent Kit (K083-3/ Bioclin, Brazil).

### Behavioral Tests

In order to analyze the animal’s free behavior we performed the open field test and two object recognition tasks: object location task (OLT) and novel object recognition task (NORT) [33–37]. The tasks were carried out in an acrylic box with three white sides and one transparent side, measuring 70 cm × 70 cm × 40 cm with a checkered bottom, which was carefully cleaned with 70% alcohol between sessions. Before the experiment began, the animal was placed in the experimental room one hour beforehand for habituation. The room had controlled lighting and temperature. As the animals are awake at night, the experiments took place after 6 pm. Care was taken to ensure silence and control odors, in order to minimize the effects of the environment. All the tests were filmed and stored for later analysis.

The tasks consist of four stages: I. Open field test (OFT); II. Training, III. Test 1 (OLT) and IV. Test 2 (NORT). OFT was carried out on the 23rd day of the experiment and training together with the tests on the 24th day of the experiment. The objects presented to the animals as stimuli were made up of plastic interlocking pieces (Gulliver, SP, Brazil) and varied in shape, color and size; they were fixed to the floor of the box so they wouldn’t move during the tests.

Open field test- the animal was allowed for two 5-min periods to freely explore the arena, separated by a period of 20 min of rest. Only the first exploration period was used to analyze the animal’s behavior. The following parameters were analyzed: total number of quadrants crossing (crossing from one quadrant to another with all four paws) and immobilization time (min).

Within 24 h after the open field test, the animal was placed back in the arena and allowed to explore two identical objects for 5 min, in a training session and then returned to their home cage. After a delay of 20 min, test session 1 (OLT) was carried out. The rats were exposed to the same objects presented in training, but one of them was in a different spatial location and was called a displaced object (DO). For test 2 (NORT) the animals returned to the arena after 20 min of the Object Location Test. The rats were placed in an arena containing a familiar object (OF) and another novel object (ON). Exploratory behavior was defined as the animal bringing its snout to approximately 2 cm from the object. Any other behavior was not considered exploratory. The results were expressed as the mean ± SEM of the Recognition Index (RI):

$$ {\text{RI }} = \frac{{\text{Time spent exploring the new object or the object in the new spatial location}}}{{\text{Total time spent exploring the two objects}}} $$

The recognition index (RI) ranges from 0 to 1.0. An RI value of 0.5 indicates equal exploration of both objects, whereas an RI significantly greater than 0.5 reflects preferential exploration of the novel object or the object in a new spatial location. An RI above 0.5 indicates that animals spent more than 50% of the total exploration time interacting with the novel or displaced object, consistent with preserved memory performance.

### Sample Preparation

For the study of biochemical parameters related to apoptosis, two neural substrates were evaluated: hippocampus and the cortex. To obtain the total extract, the samples were homogenized in a Potter-type homogenizer (MA099, Marconi), in buffer (Tris–HCl 10 mM pH 7.4, sucrose 320 mM, EDTA 0.5 mM and MgCl_2_ 1 mM, 10% protease inhibitor cocktail), at maximum speed, where samples were automatically macerated 15 to 20 times, according to the number needed to macerate all the tissue and produce as little foam as possible. The volume of buffer used was calculated by the weight of each sample following a 5:1 ratio. Total proteins were measured using the Bradford colorimetric method, with bovine serum albumin (BSA) as the standard [38].

### Western Blotting

The samples were prepared in buffer (Tris–HCl 0.125 mM pH 6.8, bromophenol blue 0.004%, 2-mercaptoethanol 10%, glycerol 20%, and SDS 4%) and distilled water. Eighty μg of proteins were applied to a 12.5% polyacrylamide gel for electrophoresis. Electrophoresis was carried out at 110 V for approximately 2 h. The proteins were then transferred to nitrocellulose membranes using the semi-dry transfer method (Trans-blot SD Semy-drytransfercell, BioRad, USA) at 15 V for 60 min. The membranes were then exposed to Ponceau red solution to check the effectiveness of the transfer. Next, the membranes were blocked with 5% skimmed milk solution for one hour and incubated with primary antibody overnight at 4 ºC, using antibodies raised against: Caspase−8 (mouse monoclonal, 1:250, catalog no. SC-81656, Santa Cruz Biotechnology, USA), Caspase−9 (mouse monoclonal, 1:250, catalog no. SC-56076, Santa Cruz Biotechnology, USA), AIF (mouse monoclonal, 1:500, catalog no. SC-13116, Santa Cruz Biotechnology, USA) and Actin (mouse monoclonal, 1:1000, catalog no. SC-81178, Santa Cruz Biotechnology, USA). Incubation with an HRP-conjugated goat anti-mouse IgG secondary antibody (1:2000, catalog no. SC-516102, Santa Cruz Biotechnology, USA) for 2 h at room temperature with shaking, followed by five 5-min washes with T-TBS (100 mM Tris-Base, 0.9% NaCl and 0.1% Tween). The immunoreactive species were visualized by chemiluminescence using a mixture of 2 solutions (solution 1: tris 100 mM pH 8.5, luminol 2.5 mM and p-coumaric acid 0.396 mM; solution 2: tris 100 mM pH 8.5 and H2O2 0.06%). The membranes were developed using a photodocumenter (L-PIX CHEMI, Loccus Biotechnology). The immunoreactive bands were quantified by densitometry using the ImageJ 1.51 program (National Institute of Health, USA). Results were expressed as the protein of interest /β-actin ratio, in relative density units. The stability of β-actin expression across the experimental groups was verified by densitometric analysis and is presented in Supplementary Figure S2.

### Statistics

Statistical analyses were performed using Prism software (version 6.0). Data distribution was assessed using the Shapiro–Wilk test. All datasets showed parametric distribution and are presented as mean ± standard error of the mean (SEM) and were analyzed using two-way analysis of variance (ANOVA), considering two independent factors: obesity status and atorvastatin treatment. Tukey’s multiple comparison test was applied as a post hoc analysis. Results were considered statistically significant at *p* < 0.05, with a 95% confidence interval.

For memory assessment, a one-sample *t*-test was employed, testing the alternative hypothesis that the sample mean differed from 0.5. A recognition index (RI) significantly greater than 0.5 indicates that animals spent more than 50% of the exploration time interacting with the novel or displaced object, reflecting preserved memory performance.

## Results

### Body Weight, Adiposity, and Cholesterol Levels

**Fig. 1:**
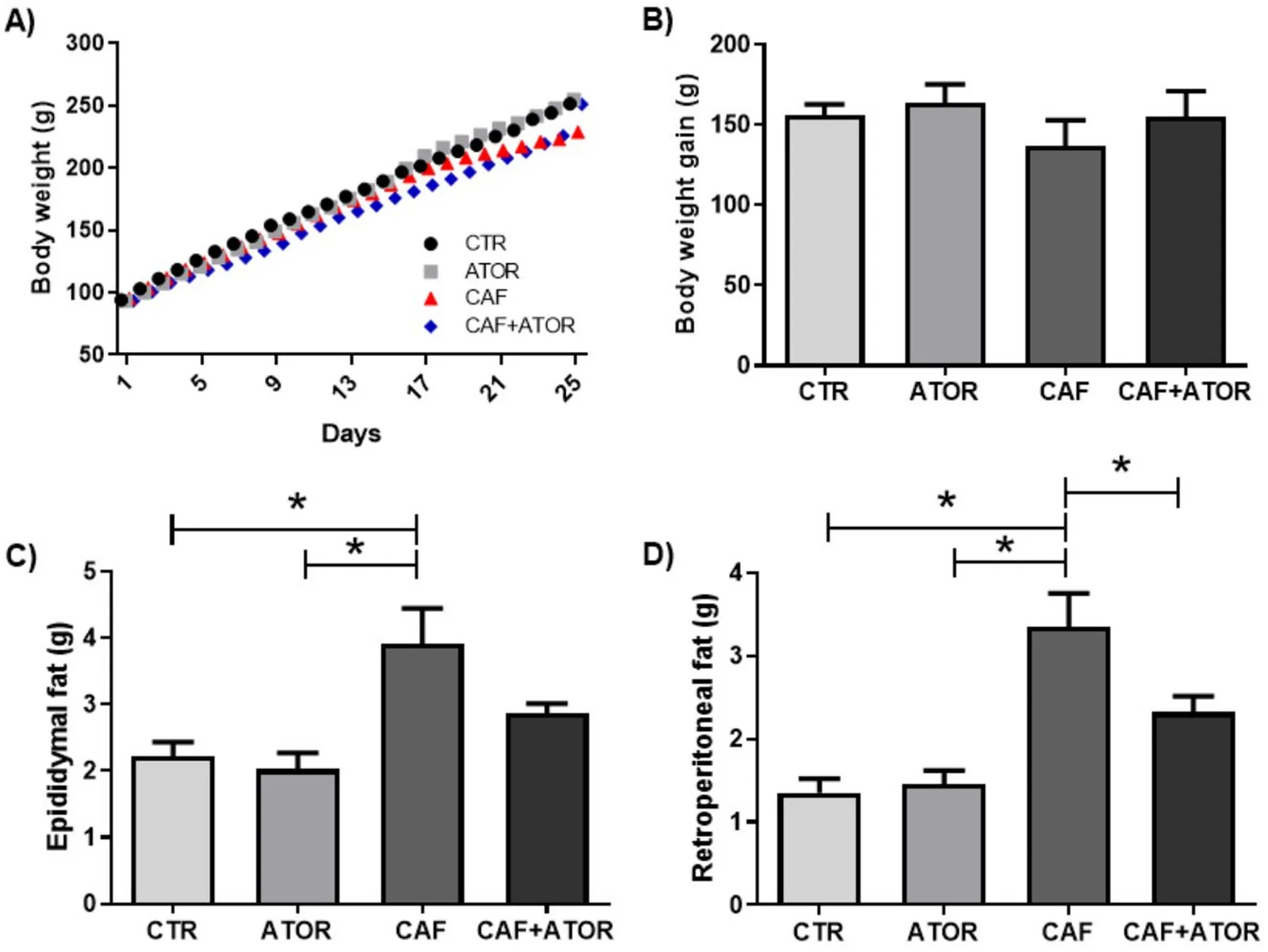
Effects of cafeteria diet feeding and atorvastatin treatment on body weight gain and adiposity in rats. **A** Time course of body weight gain during the 24-day experimental period. **B** Final body weight gain (g). **C** Epididymal adipose tissue weight (g). **D** Retroperitoneal adipose tissue weight (g). Data are presented as mean ± SEM (n = 6–8 animals per group). Statistical analyses were performed using two-way ANOVA followed by Tukey’s multiple-comparison test. *p < *0.05. CTR (standard diet), ATOR (standard diet* + *atorvastatin), CAF (cafeteria diet), and CAF*+*ATOR (cafeteria diet* + *atorvastatin)*

Consumption of the cafeteria diet and atorvastatin treatment did not induce any changes in the body weight profile of rats (Figs. 1A and B). The values obtained for final body weight gain are: CTR: 155.9 ± 6.96 g. ATOR: 163.4 ± 11.09 g. CAF: 136.6 ± 16.35 g. CAF+ATOR: 155.0 ± 16.00 g (Fig. 1B). The Two-Way ANOVA test showed no interaction between variables diet and drug (p-value 0.69).

Despite not changing total weight gain over the 24-day period evaluated, cafeteria diet-fed rats showed increased body adiposity weight of retroperitoneal (148,1%) and epididymal (82%) white adipose tissue and it was possible to observe that atorvastatin was able to reduce retroperitoneal fat in 30,8%, but no reduction in epididymal fat was detected (Figs. 1C and D). The values for epididymal fat were: CTR: 2.21 ± 0.22 g; ATOR: 2.02 ± 0.24 g; CAF: 3.92 ± 0.53 g and CAF+ATOR: 2.84 ± 0.14 g (Fig. 1C). The values for retroperitoneal fat of: CTR: 1.35 ± 0.17 g; ATOR: 1.45 ± 0.16 g; CAF: 3.35 ± 0.40 g and CAF+ATOR: 2.32 ± 0.19 g (Fig. 1D). The Two-Way ANOVA test showed that for epididymal fat the independent variables did not interact (p-value of 0.2) and for retroperitoneal fat, the test showed that there was an interaction (p-value of 0.04) of our variables with the increase in fat in the region.

**Fig. 2:**
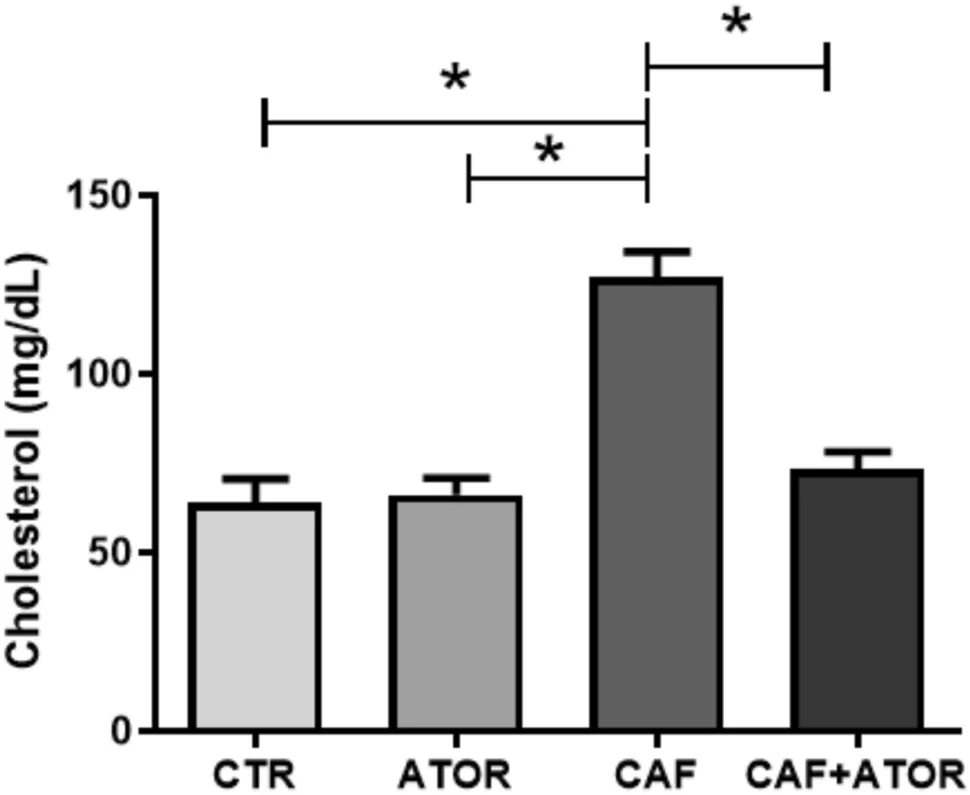
Effects of cafeteria diet feeding and atorvastatin treatment on blood cholesterol concentration in rats. Blood cholesterol concentration (mg/dL). Data are presented as mean ± SEM (n = 4 animals per group). Statistical analyses were performed using two-way ANOVA followed by Tukey’s multiple-comparison test. *p < 0.05.* CTR (standard diet), ATOR (standard diet* + *atorvastatin), CAF (cafeteria diet), and CAF*+*ATOR (cafeteria diet* + *atorvastatin)*

Cafeteria diet feeding markedly increased serum cholesterol levels (approximately 98.9% higher than the CTR group), consistent with the metabolic dysfunction induced by chronic hypercaloric/hyperlipidic feeding. Atorvastatin treatment prevented this diet-induced hypercholesterolemia, as cholesterol levels in the CAF+ATOR group were significantly lower than in the CAF group and did not differ from the CTR or ATOR groups, The values obtained were: CTR: 63.95 ± 6.66 mg/dL. ATOR: 66.09 ± 4.69 mg/dL. CAF: 127.20 ± 6.99 mg/dL. CAF+ATOR: 73.40 ± 4.81 mg/dL. The two-way ANOVA test showed a p-value for interaction of 0.0005, indicating that atorvastatin fully normalized cholesterol levels under cafeteria diet conditions (Fig. 2).

### Behavioral Analysis

**Fig. 3:**
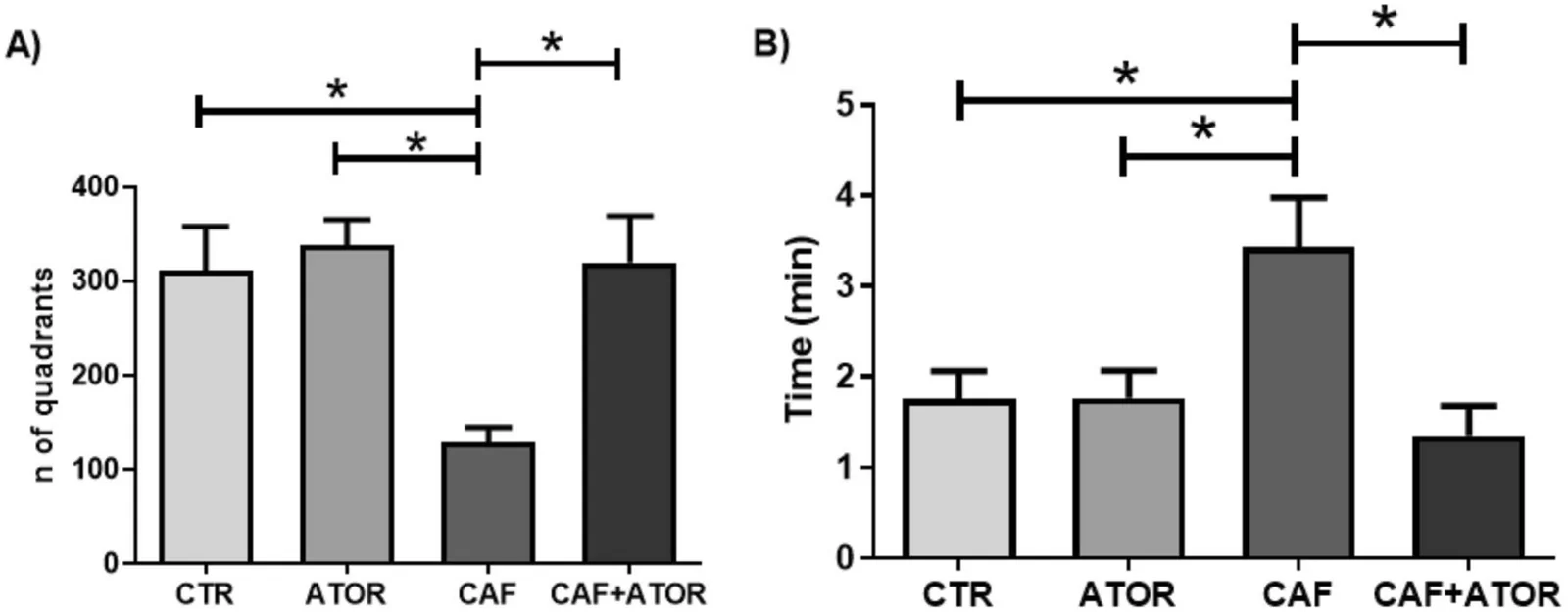
Effects of cafeteria diet feeding and atorvastatin treatment on exploratory behavior in the open field test. **A** Total crossings (number of quadrants). **B** Immobility time (s). Data are presented as mean ± SEM (n = 6 animals per group). Statistical analyses were performed using two-way ANOVA followed by Tukey’s multiple-comparison test. *p < 0.05.* CTR (standard diet), ATOR (standard diet* + *atorvastatin), CAF (cafeteria diet), and CAF*+*ATOR (cafeteria diet* + *atorvastatin)*

The cafeteria diet reduced exploratory activity by 41.4%, as indicated by a decrease in total crossings compared with controls. This impairment was effectively reversed by atorvastatin treatment. Total crossing values were 310.8 ± 47.4 quadrants in control rats, 337.3 ± 27.8 quadrants in ATOR rats, 128.7 ± 16.3 quadrants in CAF rats, and 319.8 ± 49.3 quadrants in CAF+ATOR rats (Fig. 3A).

In addition, cafeteria diet–fed rats displayed a significant increase in immobility time compared with controls, with an increase of approximately 95.3%, indicative of reduced locomotor activity. This effect was also reversed by atorvastatin treatment. Immobility times were 1.76 ± 0.30 min in control rats, 1.80 ± 0.30 min in ATOR rats, 3.40 ± 0.55 min in CAF rats, and 1.30 ± 0.30 min in CAF+ATOR rats (Fig. 3B).

**Fig. 4:**
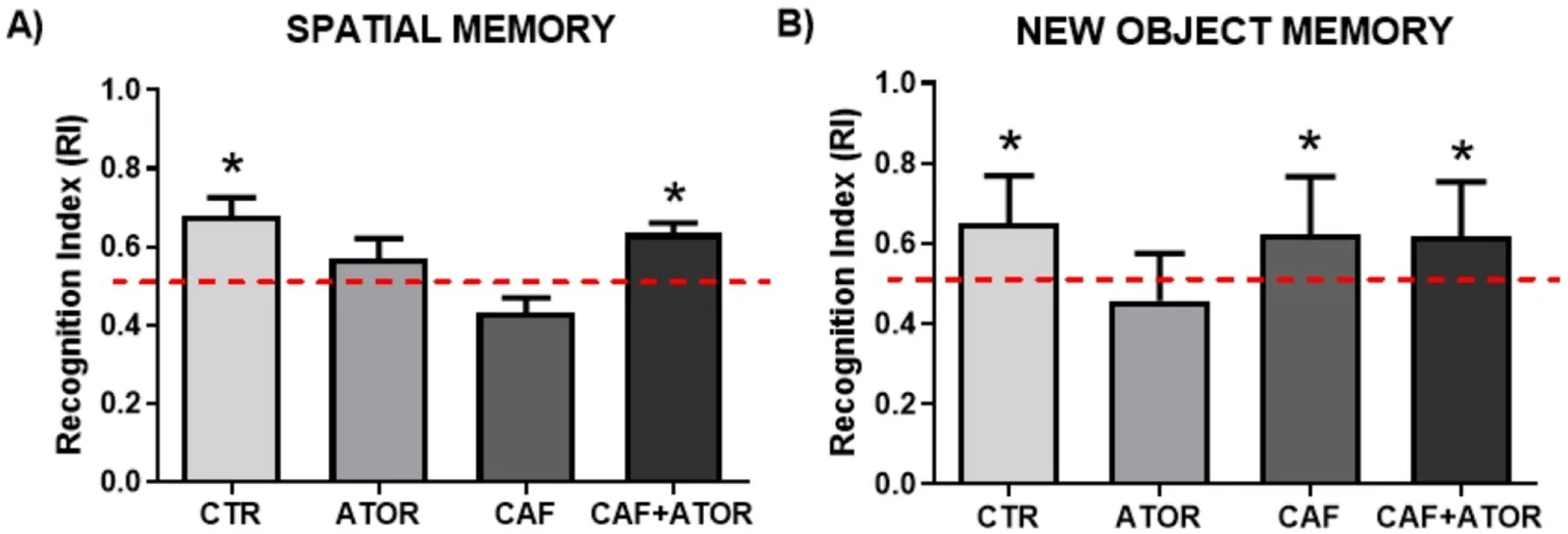
Effects of cafeteria diet feeding and atorvastatin treatment on recognition memory performance. **A** Spatial memory assessed by the Object Displacement Test (ODT). **B** Recognition memory assessed by the Novel Object Recognition (NOR) test. The red horizontal line represents the chance level (discrimination index = 0.5), indicating equal exploration of both objects. Data are presented as mean ± SEM (n = 10 animals per group). Statistical analyses were performed using a one-sample t-test against the theoretical value of 0.5. *p < 0.05.* CTR (standard diet), ATOR (standard diet* + *atorvastatin), CAF (cafeteria diet), and CAF*+*ATOR (cafeteria diet* + *atorvastatin)*

In the object displacement task (Fig. 4A), animals with preserved spatial memory preferentially explored the object placed in a new spatial location, resulting in RI values significantly greater than the reference value of 0.5, which represents equal exploration of both objects.

Control animals exhibited RI values of 0.68 ± 0.04, indicating intact spatial memory. In contrast, animals treated with atorvastatin alone (ATOR: 0.57 ± 0.05) and those fed the cafeteria diet without atorvastatin (CAF: 0.43 ± 0.04) did not differ significantly from the reference value of 0.5, suggesting impairment of spatial memory. Notably, atorvastatin administration to cafeteria diet–fed animals restored spatial memory performance (CAF+ATOR: 0.63 ± 0.02), with RI values comparable to those of the control group and significantly greater than 0.5.

Recognition memory was further assessed using the novel object recognition task (Fig. 4B). In this paradigm, preserved memory is indicated by preferential exploration of the novel object, reflected by RI values significantly greater than 0.5. Control animals (CTR: 0.65 ± 0.04), cafeteria diet–fed animals (CAF: 0.62 ± 0.05), and cafeteria diet–fed animals treated with atorvastatin (CAF+ATOR: 0.62 ± 0.04) exhibited RI values above the reference value, indicating intact recognition memory. In contrast, animals treated with atorvastatin alone did not differ significantly from 0.5 (ATOR: 0.46 ± 0.04), suggesting impairment of novel object recognition. Atorvastatin administration in cafeteria-fed animals maintained recognition memory performance at levels comparable to those observed in the cafeteria diet alone and control groups.

### Biochemical Analysis

The cafeteria diet increased expression of pro-caspase−8 and pro-caspase−9, indicating modulation of extrinsic and intrinsic apoptotic pathways. Atorvastatin treatment attenuated these alterations, resulting in caspase expression levels comparable to those observed in control animals.

**Fig. 5:**
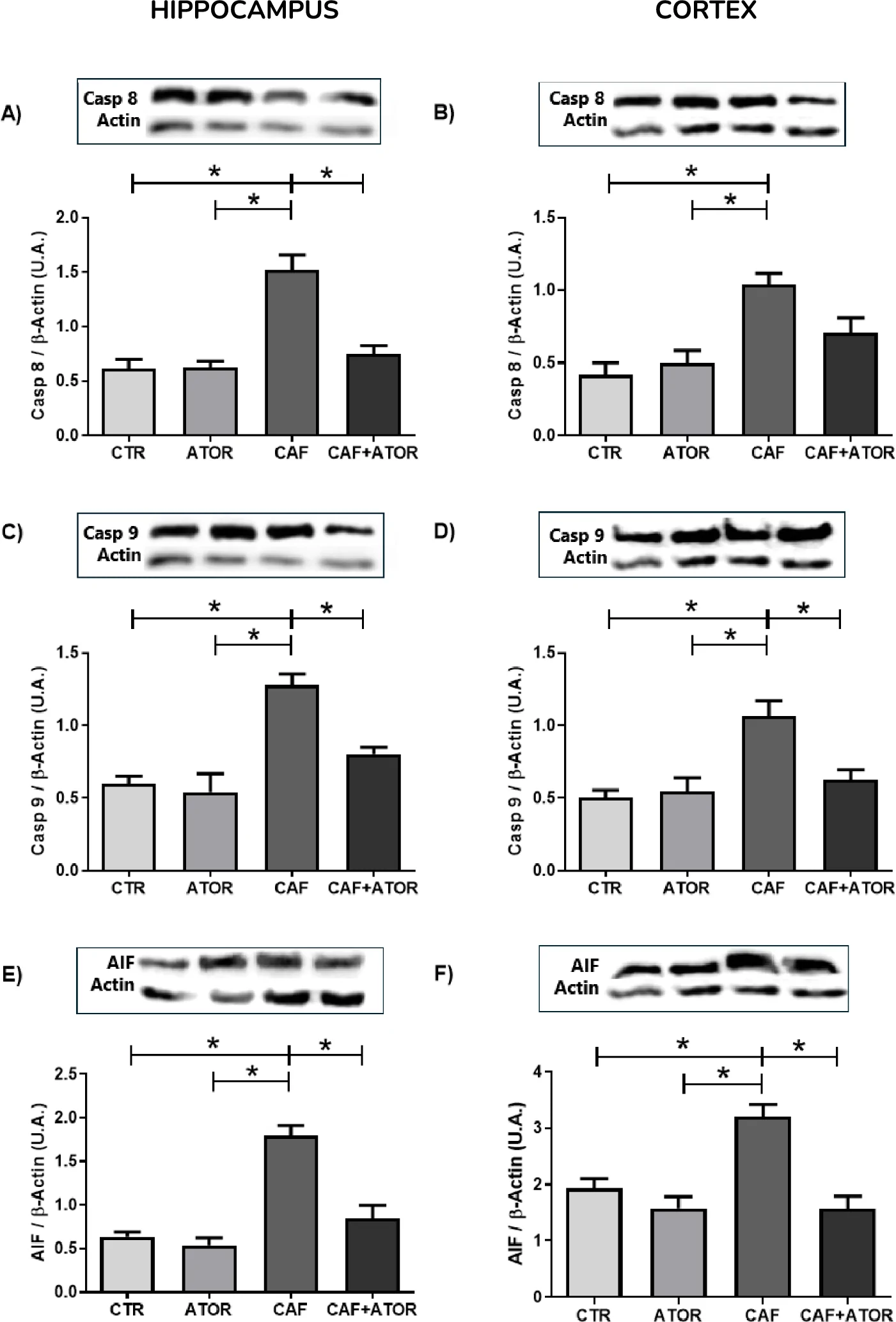
Effects of cafeteria diet feeding and atorvastatin treatment on apoptotic signaling in the hippocampus and cortex. **A**, **B** Caspase−8 protein expression in the hippocampus and cortex, respectively. **C**, **D** Caspase−9 protein expression in the hippocampus and cortex, respectively. **E**, **F** Apoptosis-inducing factor (AIF) protein expression in the hippocampus and cortex, respectively. Protein expression was normalized to β-actin. Data are presented as mean ± SEM (n = 5–6 animals per group). Statistical analyses were performed using two-way ANOVA followed by Tukey’s multiple-comparison test. *p < 0.05.* CTR (standard diet), ATOR (standard diet* + *atorvastatin), CAF (cafeteria diet), and CAF*+*ATOR (cafeteria diet* + *atorvastatin)*

In the hippocampus, caspase−8 expression was increased in cafeteria-fed rats (CAF: 1.52 ± 0.14 AU) compared with control (CTR: 0.61 ± 0.09 AU) and ATOR groups (0.62 ± 0.06 AU), while atorvastatin treatment reduced caspase−8 levels in cafeteria-fed animals (CAF+ATOR: 0.75 ± 0.07 AU) (Fig. 5A). A similar pattern was observed for hippocampal caspase−9, with increased expression in CAF animals (1.28 ± 0.08 AU) compared with CTR (0.60 ± 0.06 0.58 AU) and ATOR (0.54 ± 0.13 AU), and reduced levels following atorvastatin treatment (CAF+ATOR: 0.80 ± 0.05 AU) (Fig. 5C).

In the cortex, cafeteria-fed animals also showed elevated caspase−8 expression (CAF: 1.04 ± 0.08 AU) compared with CTR (0.41 ± 0.09 AU) and ATOR (0.49 ± 0.09 AU), which was attenuated by atorvastatin (CAF+ATOR: 0.71 ± 0.10 AU) (Fig. 5B). Cortical caspase−9 expression followed a similar trend, with increased levels in CAF animals (1.06 ± 0.11 AU) and normalization following atorvastatin treatment (CAF+ATOR: 0.62 ± 0.07 AU) compared with control values (CTR: 0.51 ± 0,05 AU and ATOR: 0.54 ± 0.09) (Fig. 5D).

Statistical analysis using the two-way ANOVA test demonstrated that cafeteria diet feeding significantly increased pro-caspase expression, whereas atorvastatin treatment partially reversed these effects, with CAF+ATOR groups showing no significant differences compared with controls. Two-way ANOVA revealed a significant interaction between diet and drug treatment, indicating that the effects of atorvastatin on caspase−8 and caspase−9 expression were dependent on dietary condition.

In addition, the expression of apoptosis-inducing factor (AIF) was assessed to further evaluate mitochondrial involvement in diet-induced apoptosis (Fig. 5E–F). Cafeteria-fed animals exhibited a marked increase in AIF expression in both the hippocampus (CAF: 1.79 ± 0.12 AU) and cortex (CAF: 3.20 ± 0.21 AU) compared with control animals (hippocampus: 0.64 ± 0.06 AU; cortex: 1.93 ± 0.17 AU). Atorvastatin treatment reduced AIF expression in cafeteria-fed animals (CAF+ATOR: 0.85 ± 0.15 AU in hippocampus; 1.57 ± 0.23 AU in cortex), resulting in values not significantly different from those observed in control and ATOR groups (hippocampus: 0.54 ± 0.82 AU; cortex: 1.57 ± 0.21 AU).

Two-way ANOVA revealed a significant interaction between diet and atorvastatin treatment for AIF expression in both brain regions, suggesting that atorvastatin attenuated diet-induced alterations in apoptosis-related signaling in the hippocampus and cortex.

## Discussion

Short-term exposure to cafeteria-style diets does not consistently produce significant increases in total body weight when compared to standard diets, particularly within experimental windows shorter than four weeks [39]. Accordingly, although total body weight gain was not significantly altered in the present study, cafeteria-fed rats exhibited clear metabolic alterations, including increased adiposity and hypercholesterolemia, confirming the induction of an obesity-like phenotype.

Previous studies demonstrate that substantial weight gain associated with cafeteria diets typically emerges after prolonged exposure, often beyond three to four weeks [40]. Our experimental timeline ended on day 24, a period during which changes in body composition precede overt body mass differences. Importantly, body weight alone is an insufficient marker of obesity, as high-fat diets preferentially promote lipid storage while reducing lean mass through disruptions in fatty acid synthesis and lipid metabolism pathways [41]. Consistent with this concept, earlier reports describe marked increases in specific adipose depots and total fat mass in the absence of significant changes in overall body weight [9, 42]. Our findings corroborate these observations, indicating that cafeteria diet–induced obesity primarily manifests as adipose tissue expansion driven by adipocyte hypertrophy and hyperplasia [43].

Atorvastatin, a competitive inhibitor of HMG-CoA reductase, is widely prescribed for the management of hypercholesterolemia [44]. In the present study, atorvastatin effectively reduced circulating cholesterol levels and prevented the increase in retroperitoneal adiposity in cafeteria-fed animals. In contrast, atorvastatin exerted minimal metabolic effects in control animals, likely due to intact homeostatic regulation of cholesterol synthesis under physiological conditions [45]. These findings reinforce the concept that the metabolic efficacy of statins is highly dependent on pre-existing metabolic imbalance [46, 47].

Behavioral analyses further corroborated the deleterious impact of the cafeteria diet on brain function. Cafeteria-fed rats displayed reduced exploratory activity and increased immobility time, behavioral patterns commonly interpreted as anxiety-like or motivational impairments in the open field test [33]. These alterations were fully reversed by atorvastatin treatment, indicating that the drug preserves locomotor and exploratory capacity in the context of metabolic dysfunction.

In contrast, the cognitive outcomes revealed a more complex, context-dependent profile. While the cafeteria diet selectively impaired spatial memory, atorvastatin administration in control animals resulted in deficits in both spatial and object recognition memory. Notably, when administered to cafeteria-fed animals, atorvastatin preserved recognition memory performance at levels comparable to controls, suggesting that its beneficial effects manifest predominantly under conditions of metabolic challenge. This dual profile supports the notion that statins exert context-dependent effects on cognitive function, influenced by baseline metabolic status.

Although atorvastatin is widely recognised for its lipid-lowering properties and its pleiotropic anti-inflammatory and antioxidant effects, its effects on cognitive function under physiological conditions remain controversial. Experimental and clinical studies have reported beneficial, neutral or even adverse cognitive outcomes, depending on the duration of treatment, the dose, the lipophilicity and the metabolic status of the participants [48, 49]. In metabolically healthy animals, in which oxidative stress and neuroinflammation are limited, atorvastatin may exert limited beneficial effects on brain function, as the pathological pathways targeted by the drug are not significantly activated. Conversely, previous studies suggest that, in conditions of metabolic dysfunction, statins may improve cognition by reducing oxidative stress, suppressing inflammatory signalling, preserving endothelial function and maintaining neuronal homeostasis, thereby producing greater benefits than those observed under physiological conditions [50]. These context-dependent actions may explain the heterogeneous cognitive effects of atorvastatin reported in the literature.

At the molecular level, the cafeteria diet induced significant alterations in apoptosis-related signaling in both the hippocampus and cortex. Increased expression of caspase−8 and caspase−9 suggests modulation of proteins associated with the extrinsic and intrinsic apoptotic pathways, respectively [51]. This coordinated alteration suggests that diet-induced metabolic imbalance engages multiple upstream death signals converging on neuronal apoptosis.

The concurrent alterations observed in proteins associated with both the intrinsic and extrinsic apoptotic pathways suggest a potential molecular interaction between these signaling cascades, rather than independent regulation. The activation of death receptors can stimulate caspase−8, which cleaves the BH3-only protein Bid into truncated Bid (tBid). Subsequently, tBid translocates to the mitochondria, promoting permeabilization of the outer mitochondrial membrane, the release of cytochrome c and the activation of caspase−9, thereby amplifying apoptotic signalling via the intrinsic pathway [52, 53]. At the same time, previous studies have shown that oxidative stress and mitochondrial dysfunction induced by the cafeteria diet may further compromise mitochondrial integrity, reinforcing apoptosis-related signaling and establishing a positive feedback loop between both pathways [54]. Therefore, the coordinated alterations observed in apoptosis-related proteins are consistent with the well-established molecular crosstalk between the extrinsic and intrinsic apoptotic pathways.

In parallel, elevated levels of apoptosis-inducing factor (AIF) were observed, indicating modulation of caspase-independent apoptotic mechanisms. AIF is released from mitochondria under conditions of oxidative stress and mitochondrial dysfunction and translocate to the nucleus, where it induces chromatin condensation and DNA fragmentation [55, 56]. Beyond its role in apoptosis, AIF is essential for maintaining mitochondrial respiratory chain integrity, and its dysregulation reflects compromised cellular bioenergetics [57]. Thus, increased AIF expression in cafeteria-fed animals suggests that metabolic stress impairs neuronal mitochondrial function, thereby promoting cell death signaling in cognition-related brain regions.

A limitation of the present study is that apoptosis was inferred from changes in the expression of apoptosis-related proteins rather than from direct measurements of apoptotic execution. Current recommendations indicate that complementary approaches, including detection of cleaved caspases, caspase activity assays, Bax/Bcl-2 ratio, cytochrome c release, TUNEL staining, or nuclear translocation of AIF, are required to better characterize and establish the functional activation of apoptotic pathways [58, 59]. Therefore, the present findings should be interpreted as evidence of modulation of apoptosis-related signaling rather than definitive activation of apoptotic pathways. Future studies incorporating these complementary approaches will be important to further elucidate the functional significance of the molecular alterations observed.

Importantly, atorvastatin treatment markedly attenuated the alterations of both caspase-dependent and caspase-independent apoptotic markers, restoring their expression to levels comparable to controls. These findings support a beneficial modulatory effect of atorvastatin under metabolically adverse conditions. Increasing evidence suggests that statins exert effects beyond lipid lowering, including modulation of oxidative stress, mitochondrial function, and inflammatory pathways [60–63]. Such mechanisms may underlie the observed normalization of apoptotic signaling in the hippocampus and cortex.

Interestingly, statins have been reported to exert both antioxidant and pro-oxidant effects depending on tissue type, exposure duration, and metabolic state. While some studies demonstrate reduced superoxide production and increased antioxidant enzyme expression [64, 65], others report increased reactive oxygen species generation and mitochondrial dysfunction [66]. The present findings suggest that, in the brain, atorvastatin may exert beneficial effects predominantly under conditions of metabolic stress, whereas its use in metabolically healthy animals may disrupt redox balance and cognitive function.

Taken together, these results demonstrate that atorvastatin exerts context-dependent effects on brain function. Under conditions of cafeteria diet–induced metabolic dysfunction, atorvastatin confers metabolic and behavioral benefits while attenuating diet-induced alterations in apoptosis-related proteins and preserving locomotor and cognitive performance. Conversely, in the absence of metabolic imbalance, atorvastatin negatively affects specific cognitive domains, highlighting potential risks associated with its use in individuals without clear metabolic indications. These findings underscore the importance of considering metabolic status when evaluating the central effects of lipid-lowering therapies and provide mechanistic insight into their dual actions on behavior and apoptosis-related molecular signaling.

Although the present findings demonstrate beneficial behavioral effects alongside modulation of apoptosis-related proteins, this study did not include direct assessment of neuronal survival, histopathological alterations, neuroinflammatory markers, or synaptic integrity, precluding a definitive conclusions regarding the relationship between alterations in apoptosis-related signaling and the observed behavioral outcomes. Future studies incorporating these complementary analyses, together with dose–response and longer-term treatment protocols, will be important to further characterize the mechanisms underlying the context-dependent effects of atorvastatin and to clarify their translational relevance.

## Supplementary Information

Below is the link to the electronic supplementary material.

Supplementary file1 (PDF 137 KB)

Supplementary file2 (PDF 128 KB)

## Data Availability

The datasets generated and analyzed during the current study are available from the corresponding author upon reasonable request.
