# Supplementary material for: Atorvastatin Exerts Context-Dependent Metabolic and Beneficial Effects on Behavior and Apoptotic Signaling in a Cafeteria Diet–Induced Metabolic Dysfunction Model
Source: Neurochem Res. 2026 Aug 29;51(5):256. doi: 10.1007/s11064-026-04874-9 (PMC13526100; doi:10.1007/s11064-026-04874-9)
Supplement: Supplementary file 1 [file 11064_2026_4874_MOESM1_ESM.pdf]

Supplementary Data N°1

ATORVASTATIN EXERTS CONTEXT-DEPENDENT METABOLIC AND NEUROPROTECTIVE EFFECTS ON APOPTOTIC SIGNALING AND BEHAVIOR IN A CAFETERIA DIET–INDUCED METABOLIC DYSFUNCTION MODEL

Ítalo Leonardo Diogo[a]; Grazielle Aparecida Silva Maia[a], Israel José Pereira Garcia[a]; Herica de Lima Santos[a]; Vanessa Faria Cortes[a]; Leandro Augusto Barbosa[a]; Valéria Ernestânia Chaves[b] and Luciana Estefani Drumond Carvalho[a]*

a. Laboratório de Bioquímica Celular, Universidade Federal de São João Del Rei, Divinópolis 35501-296, Minas Gerais, Brazil. b. Laboratório de Fisiologia, Universidade Federal de São João Del Rei, Divinópolis 35501-296, Minas Gerais, Brazil.
*Corresponding author: Luciana Estefani Drumond Carvalho: lucianadrumond@ufsj.edu.br

Table 1. Composition of palatable items present in cafeteria diet.

	**Carbohydrate (g/100g)**	**Lipid (g/100g)**	**Protein (g/100g)**	**Caloric Density (kcal/g)**
**Bacon**	**00,00**	**58,00**	**9,00**	**5,69**
**Caramel Candy**	**72,70**	**9,09**	**18,18**	**4,45**
**Cashew Nut**	**7,00**	**67,00**	**17,00**	**6,99**
**Cheese Biscuit**	**65,00**	**17,50**	**7,50**	**4,50**
**Chocolate**	**50,00**	**33,30**	**10,00**	**5,40**
**Chocolate Wafers**	**63,10**	**23,30**	**8,10**	**4,95**
**Cookies**	**63,33**	**23,33**	**3,33**	**5,00**
**Cornstarch Biscuit**	**62,50**	**12,50**	**7,50**	**4,02**
**Nougat**	**80,00**	**7,50**	**10,00**	**3,80**
**Peanut Candy**	**58,82**	**26,47**	**11,76**	**5,29**
**Potato Chips**	**45,00**	**35,00**	**5,00**	**5,15**
**Toast**	**73,33**	**6,67**	**13,33**	**4,00**
**Nuvital Feed**	**55,00**	**4,50**	**22,00**	**2,85**
