# Supplementary material for: Atorvastatin Exerts Context-Dependent Metabolic and Beneficial Effects on Behavior and Apoptotic Signaling in a Cafeteria Diet–Induced Metabolic Dysfunction Model
Source: Neurochem Res. 2026 Aug 29;51(5):256. doi: 10.1007/s11064-026-04874-9 (PMC13526100; doi:10.1007/s11064-026-04874-9)
Supplement: Supplementary file 2 [file 11064_2026_4874_MOESM2_ESM.pdf]

Supplementary Data N°2

ATORVASTATIN EXERTS CONTEXT-DEPENDENT METABOLIC AND NEUROPROTECTIVE EFFECTS ON APOPTOTIC SIGNALING AND BEHAVIOR IN A CAFETERIA DIET–INDUCED METABOLIC DYSFUNCTION MODEL

Ítalo Leonardo Diogo[a]; Grazielle Aparecida Silva Maia[a], Israel José Pereira Garcia[a]; Herica de Lima Santos[a]; Vanessa Faria Cortes[a]; Leandro Augusto Barbosa[a]; Valéria Ernestânia Chaves[b] and Luciana Estefani Drumond Carvalho[a]*

a. Laboratório de Bioquímica Celular, Universidade Federal de São João Del Rei, Divinópolis 35501-296, Minas Gerais, Brazil. b. Laboratório de Fisiologia, Universidade Federal de São João Del Rei, Divinópolis 35501-296, Minas Gerais, Brazil.
*Corresponding author: Luciana Estefani Drumond Carvalho: lucianadrumond@ufsj.edu.br

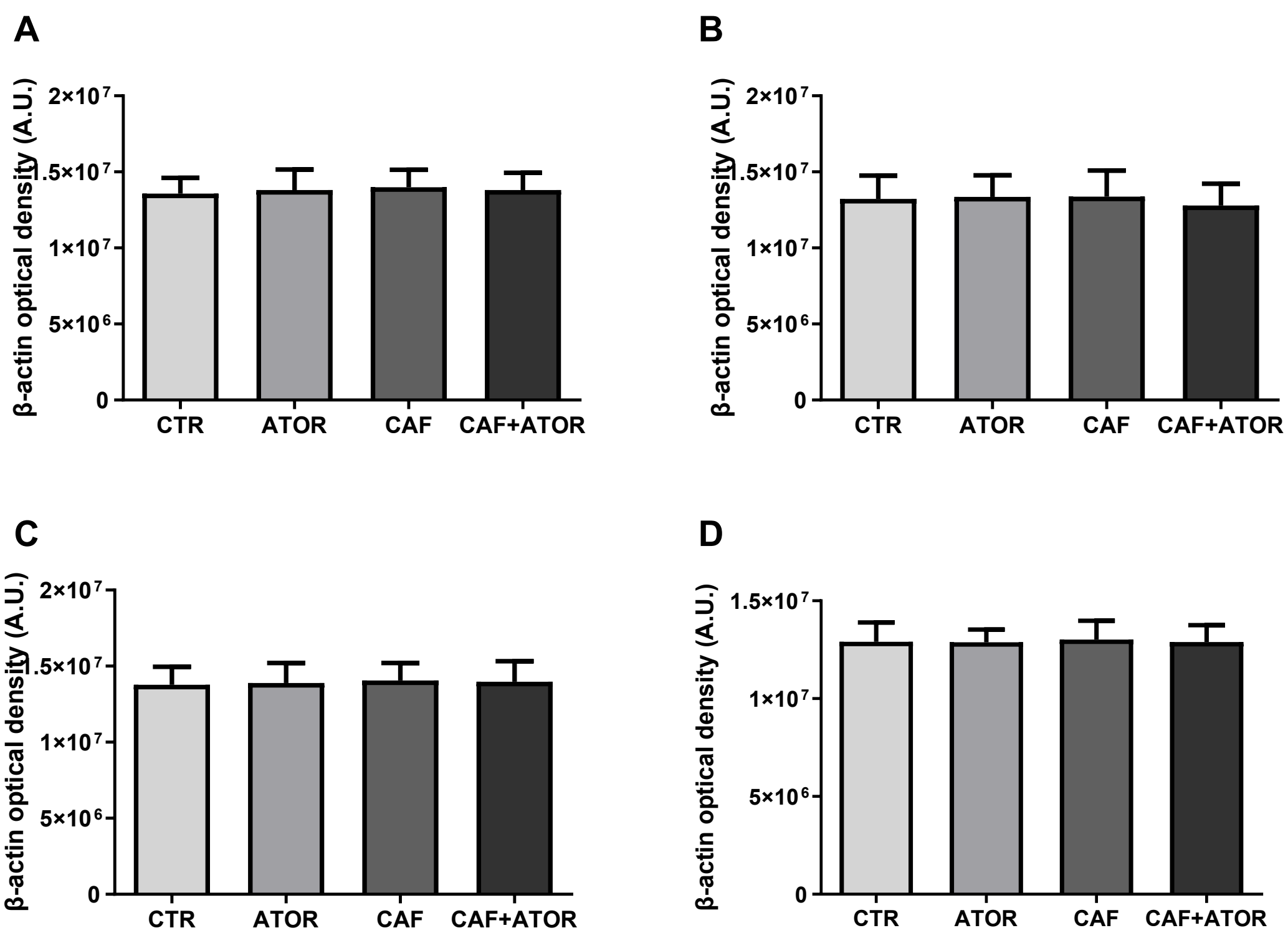

Supplementary Figure S2. Validation of β-actin stability as the loading control for Western blot normalization. Quantitative densitometric analysis of β-actin expression was performed to verify its stability across the experimental groups. **(A)** Membranes used for Caspase-8 and Caspase-9 analyses in the hippocampus. **(B)** Membranes used for Caspase-8 and Caspase-9 analyses in the cortex. **(C)** Membranes used for AIF analysis in the hippocampus. **(D)** Membranes used for AIF analysis in the cortex. Data are presented as mean ± SEM (n = 5-6 animals per group). Statistical analysis was performed using two-way ANOVA (diet × atorvastatin treatment). No significant main effects or interactions were observed ($p > 0.05$), confirming the stability of β-actin under the experimental conditions and supporting its suitability as the loading control for protein normalization. *CTR (standard diet), ATOR (standard diet + atorvastatin), CAF (cafeteria diet), and CAF+ATOR (cafeteria diet + atorvastatin).*
